# Understanding the impact of parental growth mindset on Chinese primary school students' mental health problems: the chain mediating effect of parenting self-efficacy and students' self-control

**DOI:** 10.3389/fpsyg.2025.1702114

**Published:** 2026-01-06

**Authors:** Rong-Man Yuan

**Affiliations:** 1 Students' Mental Health Center, Beijing Youth Politics College, Beijing, China; 2 Beijing Key Laboratory of Applied Experimental Psychology, Faculty of Psychology, Beijing Normal University, Beijing, China

**Keywords:** chain mediating effect, mental health, parental growth mindset, parenting self-efficacy, self-control

## Abstract

**Purpose:**

The mental health of primary school students in China has garnered significant attention. Various factors can influence the mental health of primary school students, with family-related factors being particularly important. This study aims to explore the relationship between parental growth mindset and primary school students' mental health problems, as well as the longitudinal mediating mechanisms of parenting self-efficacy and students' self-control in this relationship. By providing theoretical support and practical guidance, this study aims to improve the mental health of primary school students.

**Methods:**

This study utilized a three-wave longitudinal design spanning 1 year, recruiting students and parents from two primary schools in Beijing. Parental growth mindset (T1) and parenting self-efficacy (T2) were assessed via parent self-report using Parental Growth Mindset Scale and Parenting Self-Efficacy Scale. Students' self-control (T2) and mental health problems (T3) were assessed via student self-report using the Brief Self-Control Scale and the Strengths and Difficulties Questionnaire. Data were collected in three waves, resulting in 280 valid matched questionnaires.

**Results:**

Parental growth mindset significantly negatively predicted mental health problems in primary school students (β = −0.25, SE = 0.02, *p* < 0.001, 95% CI [−0.129, −0.049]). Parental growth mindset can influence mental health problems in primary school students through parenting self-efficacy (effect value = −0.09, 95% CI [−0.152, −0.036]), self-control (effect value = −0.04, 95% CI [−0.083, −0.005]), and chain mediating effects of parenting self-efficacy and students' self-control (effect value = −0.01, 95% CI [−0.030, −0.002]).

**Conclusion:**

Parenting self-efficacy and students' self-control play a chain mediating role between parental growth mindset and students' mental health problems in primary school students. This conclusion provides suggestions for improving mental health in primary school students.

## 1 Introduction

Mental health issues are on the rise among students and are impacting younger age groups. According to a global report by the World Health Organization (WHO), approximately 14% of mental health problems occur during childhood and adolescence. In China, the mental health of primary school students has become a key area of focus (Li et al., 2022). A meta-analysis examining the detection rates of mental health problems among primary and middle school students in China indicates that the prevalence of mental health issues ranges from 3.6 to 25.2% (Huang et al., 2022). Once they emerge, these mental health problems often persist into adulthood (Conway et al., 2017) and are associated with adverse outcomes (Kopp et al., 2024) such as long-term mental health challenges (Andersen and Gunes, 2018; Jonsson et al., 2011). Consequently, investigating the factors and mechanisms underlying mental health in primary school students is of great practical importance.

## 2 Review of the literature

### 2.1 Theoretical perspective

This study integrates Developmental Cascade Theory (Masten and Cicchetti, 2010) and Social Cognitive Theory (Bandura and National Institute of Mental Health, 1986) to explain how parental influences shape children's mental health. Developmental Cascade Theory posits that human development involves continuous reciprocal influences among multiple factors, wherein each developmental state depends on preceding states and influences subsequent ones (Bernier et al., 2020). This principle provides the theoretical foundation for examining how parental factors influence students' later mental health outcomes. Social Cognitive Theory enhances this framework by explaining how transmission occurs. Parental thoughts directly influence parenting behaviors, which serve as strong models for children's learning; through observational learning, children develop skills that later affect their development.

### 2.2 The relationship between parental growth mindset and students' mental health problems

Growth mindset refers to the belief that abilities can be developed and improved through effort and the guidance of others (Dweck and Yeager, 2019). Parental growth mindset is the belief that a child's ability can be developed through effort and guidance. This study focuses on parental growth mindset, as parents are the most influential figures in students' socialization process (Bronfenbrenner and Morris, 2006) and their thought patterns exert crucial influence on students' mental health (Ohtani et al., 2020). Prior research has demonstrated that parental growth mindset in the domain of intelligence can influence students' learning quality (Huang et al., 2023), academic performance (Guo, 2025), and positive behaviors (Garibaldi et al., 2020; Haimovitz and Dweck, 2016; Jose and Bellamy, 2012; Schleider et al., 2016). However, growth mindset is a concept of domain-specific, meaning individuals may exhibit varying growth mindset tendencies across different areas. While parental growth mindset has been linked to children's academic and behavioral outcomes, the extent to which parental growth mindset specifically targeting self-control impacts students' mental health remains unexplored. This gap is particularly salient because self-control deficits are crucial to many childhood mental health problems (He et al., 2023; Nedelec and Beaver, 2014), yet no longitudinal research has tested whether parental growth mindset of self-control serve as a protective factor. This research aims to examine the impact of parental growth mindset on the mental health problems of primary school students and uncover its underlying mechanisms.

### 2.3 The mediating effect of parenting self-efficacy

Parenting self-efficacy reflects parents' confidence in their ability to fulfill their parental role effectively and positively influence their children's development (Kendall and Bloomfield, 2005), representing an application of self-efficacy in the parenting context. Research has indicated that parents who possess a growth mindset in specific areas, such as mathematics and reading, generally exhibit higher parenting self-efficacy (Muenks et al., 2015). This enhanced sense of self-efficacy as parents leads to greater confidence in their ability to support their children's development. Additionally, numerous studies have established a link between parenting self-efficacy and children's mental health outcomes (Ahun et al., 2018; Albanese et al., 2019), with higher levels of parenting self-efficacy correlating with fewer behavioral problems and improved psychological wellbeing in children (Albanese et al., 2019; Fang et al., 2021). Based on these findings, this study proposes that parenting self-efficacy serves as a mediator in the relationship between parental growth mindset and mental health problems among primary school students.

### 2.4 The mediating effect of students' self-control

The potential mediating role of students' self-control in the relationship between parental growth mindset and students' mental health was also investigated in this study. Research indicates that parental mindsets are pivotal in shaping children's educational outcomes (Guay et al., 2024). Notably, children of parents who embrace a growth mindset tend to display better self-regulation (Stern and Hertel, 2020), directly linking parental mindset to students' capabilities. This connection is further supported by evidence indicating that parental growth mindset positively predicts children's academic achievement (Matthes and Stoeger, 2018) and is closely associated with enhanced reading abilities (Song et al., 2022), suggesting a broader enhancement of students' abilities. Self-control, defined as the ability to act voluntarily toward long-term goals despite conflicting impulses, has been associated with numerous positive long-term outcomes (Duckworth and Steinberg, 2015; Nigg, 2017), particularly in physical and mental health. Previous studies have highlighted the critical role of self-control in students' adaptation and development (Duckworth, 2011). Individuals with high levels of self-control are significantly less likely to experience mental health problems than people with low levels of self-control (de Ridder et al., 2012; Fergusson et al., 2013), thus highlighting self-control as a protective factor for mental health (Kim et al., 2022). Based on these established associations, this study suggests that self-control may mediate the relationship between parental growth mindset and students' mental health. Specifically, it is hypothesized that the positive effects of parental growth mindset on students' mental health problems are partly mediated by enhanced self-control, offering a potential pathway for interventions aimed at improving mental health outcomes in primary school students.

### 2.5 The chain mediating role of parenting self-efficacy and students' self-control

According to Developmental Cascade Theory, effects propagate across developmental domains, such that parent-level factors shape proximal parenting processes, which subsequently cultivate child-level competencies that influence distal outcomes (Masten and Cicchetti, 2010). According to social cognitive theory, children acquire behaviors and attitudes by observing and imitating their parents (Wang et al., 2007). Parents with higher levels of parenting self-efficacy are more likely to instill confidence in their children's abilities and may provide a supportive environment. This supportive environment not only encourages children to develop confidence in their own abilities but also promotes the development of abilities such as self-control. Previous research has demonstrated a strong association between parenting self-efficacy and various aspects of child behavior, including self-control (Albanese et al., 2019). Given these findings, it is plausible to propose that parenting self-efficacy is linked to self-control in primary school students. It is hypothesized that parental growth mindset can indirectly influence students' mental health problems through the chain mediation effect of parenting self-efficacy and students' self-control.

### 2.6 The present study

**Figure 1:**
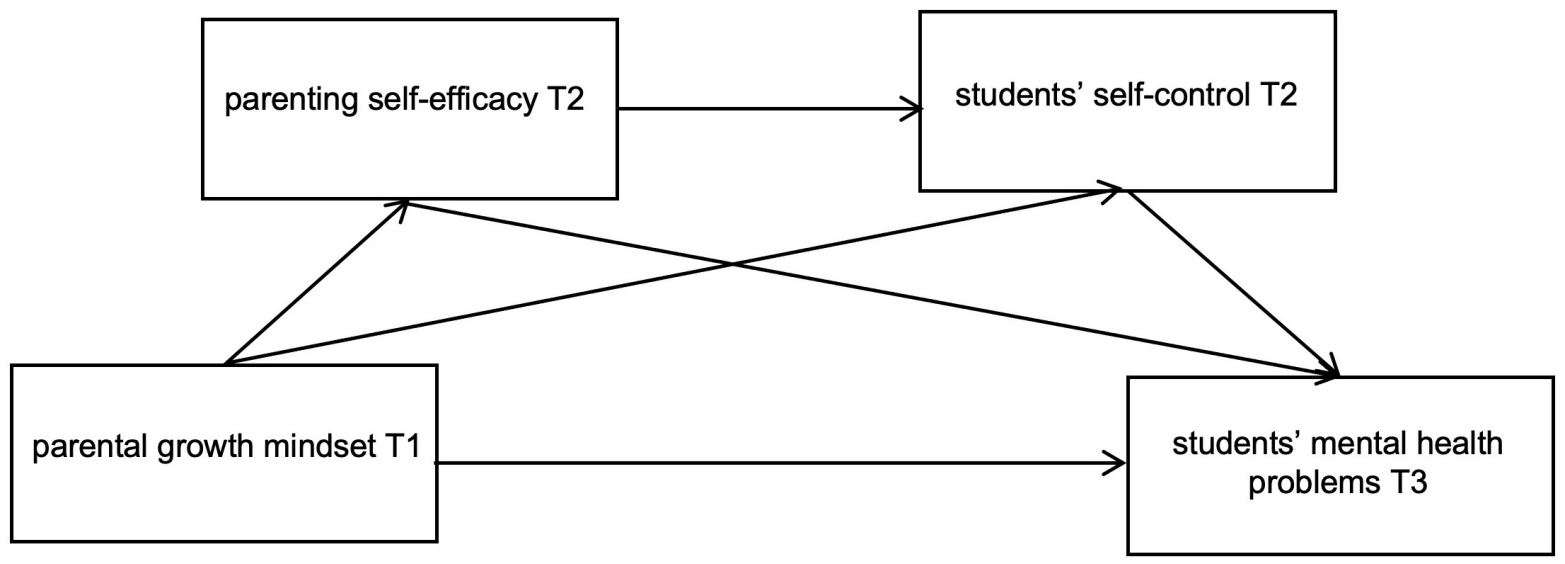
Presents the hypothesized model. T1, time 1; T2, time 2; T3, time 3.

The current longitudinal investigation integrates Developmental Cascade Theory and Social Cognitive Theory to test a sequential mediation model (Figure 1). This model posits that parental growth mindset (T1) indirectly influences primary school students' mental health problems (T3) through the following pathways: (a) parenting self-efficacy (T2); (b) students' self-control (T3); and (c) the chain mediation pathway of parenting self-efficacy and students' self-control. The specific hypotheses guiding this research include:

H1: Parental growth mindset (T1) will negatively affect mental health problems (T3) in primary school students.
H2: Parenting self-efficacy (T2) will mediate the relationship between parental growth mindset (T1) and students' mental health problems (T3).
H3: Students' self-control (T2) will mediate the relationship between parental growth mindset (T1) and students' mental health problems (T3).
H4: Parenting self-efficacy (T2) and students' self-control (T2) will sequentially mediate the relationship between parental growth mindset (T1) and students' mental health problems (T3).

## 3 Methodology

### 3.1 Participants

The study utilized a convenience sampling method, recruiting participants from two public schools in Beijing's urban area. This school's comprehensive 9-year system, spanning primary to high school, provided a stable and robust participant pool, making it ideal for longitudinal research. Using cluster sampling, the study targeted students from grades 3 to 6, a critical developmental stage as highlighted by previous research (Molden and Dweck, 2006). At the first time point in March 2024 (T1), parental growth mindset data were collected, with 345 parent questionnaires gathered. At the second time point (T2) in September 2024, parenting efficacy data and students' self-control questionnaires were collected, yielding 360 student and 320 parent questionnaires. At the third time point (T3), in March 2025, students' strengths and difficulties data were collected, and a total of 356 questionnaires were returned. Data from students and parents who completed all three waves of data collection were retained, resulting in 280 valid matched questionnaires. Using the simsem package in R (Schoemann et al., 2017), we performed Monte Carlo simulations with 10,000 replications to estimate statistical power for the two-serial-mediator model. Employing the Monte Carlo confidence interval method with the objective of determining power at a fixed sample size (*N* = 280) and assuming low inter-variable correlations (*r* = 0.28), power to detect the indirect effect exceeded.80, demonstrating that our sample size was adequate for detecting the hypothesized mediation pathways. The student sample consisted of 157 boys and 123 girls, with a mean age of 10.56 years (SD = 0.95). Specifically, there were 113 students in the third grade, 84 in the fourth grade, and 83 in the fifth grade. Among the parents, 70 fathers and 210 mothers participated, with a mean age of 40.01 years (SD = 3.63). Regarding educational background, one parent had lower-secondary education or below, nine had upper-secondary or vocational education, 33 had a community college or associate degree, 185 had a bachelor's degree, and 52 had a master's degree or higher.

### 3.2 Procedure

This study was approved by the Ethics Committee of Beijing Normal University (BNU202311010167) and employed a three-wave longitudinal design with a 6-month interval between each wave. The research was conducted by graduate psychology students who received standardized training to ensure the research process was both normative and professional. First, researchers introduced recruitment letters to the school and asked teachers to distribute them to students. Then, they obtained written informed consent from the parents. Data were collected at three time points: at the first time point in March 2024 (T1), parents reported their growth mindset and demographic information via questionnaires distributed through a WeChat parent group using a “Wenjuanxing” link. At the second time point in September 2024 (T2), parents' parenting self-efficacy questionnaires and students' self-control data were collected. Researchers took students to the school's computer room during the lunch break to complete the student questionnaires via a “Wenjuanxing” link on the computer. Parent questionnaires were distributed following the same procedure as in T1. At the third time point in March 2025 (T3), the students' Strengths and Difficulties Questionnaire was administered following the same procedure as in T2. To ensure data validity, questionnaires completed in less than 5 s (indicative of random responding) were excluded from the primary analysis (*n* = 4). Sensitivity analyses were performed by re-estimating the chain mediation model with (*n* = 284) and without (*n* = 280) these cases. All indirect effects remained statistically significant, confirming the robustness of our results. The data of students and parents were matched using identification numbers collected during the data collection process. This matching process allowed for the longitudinal tracking of the relationship between parental variables and student variables.

### 3.3 Measures

#### 3.3.1 Parental growth mindset

Parental growth mindset was assessed using a modified version of the Growth Mindset Scale (Dweck et al., 1995), In line with prior studies, the core concept of “intelligence” was replaced with “self-control” to suit the study's context better (Matthes and Stoeger, 2018), and items were rephrased to refer to “my child” instead of “I.” The adapted scale consisted of six items, each rated on a 6-point Likert scale, ranging from 1 (strongly disagree) to 6 (strongly agree). For example, “My child's self-control is something about which he/she can't change very much” (reversed-coded). After recoding the reverse-scored items, the average score was computed based on the total of all items. Higher scores suggest a higher level of parental growth mindset. Prior studies have confirmed the scale's good reliability and validity among Chinese participants, including both children (Yuan et al., 2024) and parents (Huang et al., 2023). In the current study, the scale demonstrated good reliability at T1 with a Cronbach's alpha coefficient of 0.84 and the coefficient omega (ω) of 0.84. The confirmatory factor analysis indicated acceptable construct validity for the parental growth mindset: χ^2^/df = 0.451, RMSEA = 0.000, CFI = 1.000, TLI = 1.000, SRMR = 0.007.

#### 3.3.2 Parenting self-efficacy

Parenting self-efficacy was measured using a scale adapted from Hoover-Dempsey et al. (1992), following the domain-specific adaptation procedure outlined by Muenks et al. (2015). The five items assess parents' perceived efficacy in fostering their child's self-control abilities. This scale was used to assess parents' perceived efficacy in helping their child with self-control. Parents rated five items on a six-point Likert scale, ranging from 1 (“strongly disagree”) to 6 (“strongly agree”). Reverse-scored items were recoded, and the average of all items' scores was calculated to reflect parenting self-efficacy, with higher scores indicating greater parenting self-efficacy. In this study, the scale showed good reliability at T2 with a Cronbach's alpha coefficient of 0.75 and the coefficient omega (ω) of 0.73.

#### 3.3.3 Self-control

Self-control was assessed using the revised version of the Self-Control Scale, adapted for Chinese students (Unger et al., 2016). This 13-item Brief Self-Control Scale, derived from the original scale by Tangney, Baumeister, and Boone (Tangney et al., 2004), utilizes a 5-point rating scale (1 = “not at all,” 5 = “very much”). For example, “I am good at resisting temptation.” Higher scores indicate greater self-control capacity. When applied to Chinese primary school students, the scale demonstrates good validity and reliability (Jie et al., 2024). In this study, the scale had good reliability at T2 with a Cronbach's alpha coefficient of 0.79 and the coefficient omega (ω) of 0.80.

#### 3.3.4 Mental health problems

Mental health problems were assessed using the Strengths and Difficulties Questionnaire (SDQ; Goodman, 2001), developed by Goodman. The SDQ comprises five scales: emotional symptoms (five items), peer relationship problems (five items), conduct problems (five items), hyperactivity/inattention (five items), and prosocial behavior (five items). Following previous research (Shi et al., 2023), we calculated the average scores for internalizing problems (emotional symptoms and peer relationship problems scales) and externalizing problems (conduct problems and hyperactivity/inattention scales). In the current study, the SDQ was completed by students. The total mental health problems score was the average of these two scores, with higher values indicating more severe mental health problems. The scale is widely used in China and has demonstrated good reliability and validity (Du et al., 2008). In this study, the scale was administered at T3, yielding a Cronbach's alpha coefficient of 0.82 and the coefficient omega (ω) of 0.72.

### 3.4 Data analysis

All statistical analyses were performed using SPSS 29.0 and the PROCESS 4.2 macro. Initially, descriptive statistics were calculated. Next, partial correlation analysis was conducted to assess the relationships between the variables. Finally, all variables were calculated using Model 6 in PROCESS v4.2. In this model, parental growth mindset was treated as the independent variable, students' mental health problems as the dependent variable, parenting self-efficacy and students' self-control as the mediating variables, and parental education level as the control variable. The bootstrap method with 5,000 repetitions was used to test the indirect effects. An indirect effect was considered significant if its 95% confidence interval did not include 0.

## 4 Results

### 4.1 Common method bias analysis

Harman's single-factor test was used to assess common method bias. The results showed that there were 13 factors with eigenvalues greater than 1. The first factor accounted for 16.20% of the variance, below the 40% threshold (Podsakoff et al., 2003), indicating that there was no significant common method bias in this study. Critically, our design includes temporal separation (6-month intervals), rater source separation (parents vs. students), and procedural separation (home vs. school), which collectively minimize common method bias.

### 4.2 Model diagnostics

Model diagnostics were performed to verify multicollinearity. Diagnostic analyses indicated that all VIFs were below 2.0 (range: 1.13–1.26), well below the conventional threshold of 10, indicating no multicollinearity.

### 4.3 Descriptive statistics and correlation analysis

Table 1 presents the means, standard deviations, and correlation coefficients for parental growth mindset (T1), parenting self-efficacy (T2), students' self-control (T2), and students' mental health problems (T3). Correlation analysis revealed that parental growth mindset (T1) was positively associated with parenting self-efficacy (T2) (*r* = 0.41, *p* < 0.01) and students' self-control (T2) (*r* = 0.29, *p* < 0.01), and was also negatively correlated with students' mental health problems (T3) (*r* = −0.26, *p* < 0.01). Parenting self-efficacy (T2) was positively correlated with students' self-control (T2) (*r* = 0.28, *p* < 0.01) and negatively correlated with students' mental health problems (T3) (*r* = −0.31, *p* < 0.01). Furthermore, students' self-control (T2) was negatively correlated with students' mental health problems (T3) (*r* = −0.28, *p* < 0.01).

**Table 1:** Descriptive statistics and correlations among major variables.

**Variable**	**1**	**2**	**3**	**4**
1. Parental growth mindset T1	–			
2. Parenting self-efficacy T2	0.41^**^	–		
3. Students' self-control T2	0.29^**^	0.28^**^	–	
4. Students' mental health problems T3	−0.26^**^	−0.31^**^	−0.28^**^	–
*M*	4.41	3.64	3.90	1.54
SD	0.88	0.57	0.61	0.31

T1, time 1; T2, time 2; T3, time 3.

^**^p < 0.01.

### 4.4 Chain-mediated analysis of parenting self-efficacy and students' self-control

**Figure 2:**
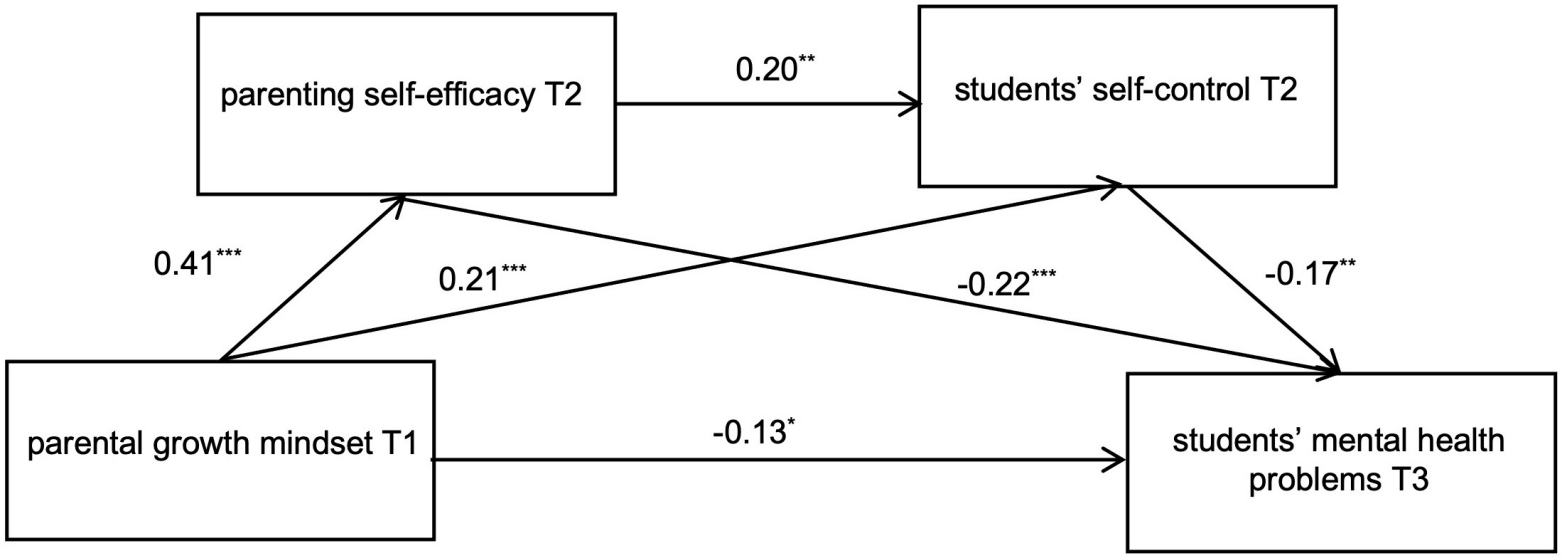
Diagram of the chain mediating effect model. T1, time 1; T2, time 2; T3, time 3. *p < 0.05, **p < 0.01, ***p < 0.001.

A mediation analysis was conducted using SPSS PROCESS Model 6, with parental growth mindset as the independent variable, primary school students' mental health problems as the dependent variable, parenting self-efficacy and students' self-control as mediators, and parental education level as the control variable. The results, presented in Table 2 and Figure 2, show that parental growth mindset significantly negatively predicts mental health problems in primary school students (β = −0.27, SE = 0.02, *p* < 0.001, 95% CI [−0.133, −0.054]). When the mediators were introduced, parental growth mindset was found to positively predict both parenting self-efficacy (β = 0.41, SE = 0.04, *p* < 0.001, 95% CI [0.198, 0.339]) and students' self-control (β = 0.21, SE = 0.04, *p* < 0.001, 95% CI [0.083, 0.342]). In addition, the direct effect of parental growth mindset on students' mental health problems became significant (β = −0.13, SE = 0.02, *p* < 0.05, 95% CI [−0.087, −0.002]). Parenting self-efficacy had a positive effect on students' self-control (β = 0.20, SE = 0.07, *p* < 0.01, 95% CI [0.083, 0.342]) and a negative effect on students' mental health problems (β = −0.22, SE = 0.03, *p* < 0.001, 95% CI [−0.182, −0.052]). Furthermore, students' self-control negatively predicted students' mental health problems (β = −0.17, SE = 0.03, *p* < 0.01, 95% CI [−0.143, −0.027]).

**Table 2:** Analysis results of the chain mediation model of parental growth mindset on students' mental health problems.

**Regression equation**		***R* ^2^**	***F***	**Significance level**	
**Dependent variable**	**Independent variable**			β	***t***
Parenting self-efficacy T2	Parental education level	0.17	28.69^***^	0.01	0.27
	Parental growth mindset T1			0.41	7.51^***^
Students' self-control T2	Parental education level	0.12	12.85^***^	−0.08	−1.37
	Parental growth mindset T1			0.21	3.45^***^
	Parenting self-efficacy T2			0.20	3.22^**^
Students' mental health problems T3	Parental education level	0.17	13.79^***^	0.15	2.65^**^
	Parental growth mindset T1			−0.13	−2.07^*^
	Parenting self-efficacy T2			−0.22	−3.56^***^
	Students' self-control T2			−0.17	−2.87^**^

T1, time 1; T2, time 2; T3, time 3.

^*^p < 0.05.

^**^p < 0.01.

^***^p < 0.001.

Bootstrap analysis with 5,000 samples revealed significant total indirect effects (95% CI does not include 0), confirming the significant mediating roles of parenting self-efficacy and students' self-control in the relationship between parental growth mindset and students' mental health. Specifically, three pathways demonstrated significant mediation: parental growth mindset → parenting self-efficacy → students' mental health problems (effect value = −0.09, 95% CI [−0.156, −0.037]); parental growth mindset → students' self-control → students' mental health problems (effect value = −0.04, 95% CI [−0.083, −0.003]); parental growth mindset → parenting self-efficacy → students' self-control → students' mental health problems (effect value = −0.01, 95% CI [−0.028, −0.001]). These pathways are detailed in Table 3.

**Table 3:** Results of the bootstrap method of the mediating effect test.

**Mediation model paths**	**Effect**	**SE**	**Bootstrap 95% CI**
Parental growth mindset T1 → parenting self-efficacy T2 → students' mental heath problems T3	−0.09	0.03	[−0.156, −0.037]
Parental growth mindset T1 → students' self-control T2 → students' mental heath problems T3	−0.04	0.02	[−0.083, −0.003]
Parental growth mindset T1 → parenting self-efficacy T2 → students' self-control T2 → students' mental heath problems T3	−0.01	0.01	[−0.028, −0.001]
Total indirect effect	−0.14	0.03	[−0.214, −0.077]

T1, time 1; T2, time 2; T3, time 3.

## 5 Discussion

The present study employed a longitudinal design to investigate the relationship between parental growth mindset and the mental health problems of primary school students. It also explored the chain mediating roles of parenting self-efficacy and students' self-control. The findings offer valuable insights and suggestions for enhancing the mental health of primary school students, contributing to the existing literature in this field and providing practical implications for educational settings. The most important theoretical contribution of this study is framing students' self-control as the proximal mediator through which distant parental factors cascade to influence students' mental health. Consistent with developmental cascade theory (Masten and Cicchetti, 2010), our findings reveal a temporally ordered process: parental growth mindset (distant cognition) → parenting efficacy (first-stage mediator) → students' self-control (proximal mediator) → mental health outcomes (ultimate adaptation). Our domain-specific operationalization measures both parental growth mindset and parenting efficacy specifically in the context of self-control, thereby positioning self-control as the final link transmitting parental influences to mental health. The following sections elaborate on how each pathway uniquely supports this cascade framework.

Our study found that parental growth mindset significantly and negatively predicts mental health problems in primary school students. Importantly, this effect is fundamentally channeled through the self-control domain; when parents possess a growth mindset about developing their children's self-control, they are more likely to develop confidence in fostering these abilities. Specifically, higher levels of parental growth mindset were associated with fewer mental health problems in primary school students, consistent with prior studies. Recent research has increasingly examined the effect of parents' mindsets on their children, revealing that parental mindset is a key factor in shaping students' behaviors (Justice et al., 2020). Our findings may expand the ecological systems theory (Bronfenbrenner and Morris, 2006), which identifies the family as the most direct microsystem influencing an individual's psychological development. Previous studies have emphasized the influence of family atmosphere (Li and Li, 2025), parenting behaviors, and parenting attitudes on children's mental health (Cohrdes and Göbel, 2022; Qi and Wu, 2024). Moreover, our study suggests that parents' beliefs in their children's abilities also play a role, confirming that parental growth mindset serves as a positive protective factor for primary school students' mental health. Overall, these findings provide a valuable foundation for exploring family-related factors that can enhance students' mental health.

The analysis of mediating effects revealed that parental growth mindset can influence the mental health of primary school students through parenting self-efficacy. When parents possess a growth mindset regarding their children's self-control abilities, they are more likely to develop confidence in fostering these abilities (Huang et al., 2023). This aligns with prior research, which indicates that parents who view their children's abilities as malleable are more confident in facilitating their development. Consequently, these parents are more likely to persevere when faced with parent-child conflicts (Huang et al., 2023). Moreover, parents who believe in their capacity to positively influence their children are more likely to engage in positive parenting behaviors. Such behaviors can reduce children's stress and anxiety, promote their positive adaptation (Ardelt and Eccles, 2001), and ultimately enhance their mental health (Jones and Prinz, 2005). This finding is consistent with previous studies that have demonstrated the importance of parenting self-efficacy in shaping effective parenting practices and promoting positive child outcomes. Overall, the current study contributes to the understanding of how parental mindsets and self-efficacy can jointly influence primary school students' mental health problems.

In addition to the pathway through parenting self-efficacy, the present longitudinal findings indicated that parental growth mindset also influences primary school students' mental health via the promotion of self-control. Specifically, parents who adopt a growth mindset toward their children's self-control are more likely to exhibit autonomy-supportive behaviors. These behaviors, such as setting incremental goals, modeling coping strategies, and offering process-focused feedback, have been empirically linked to enhanced self-control in children. Consistent with prior studies showing that parents' incremental beliefs about intelligence foster children's language development (Muenks et al., 2015), our results extend this pattern to self-control. Parents with a growth mindset are more likely to view self-control challenges as learning opportunities, thereby persisting in efforts that strengthen their children's self-control. Previous research has indicated that parental behaviors during child-rearing significantly influence children's development. According to intergenerational transmission theory, parental traits—including personality, values, attitudes, beliefs, and behaviors—are gradually transmitted to offspring (Bowles et al., 2009; Chen and Liu, 2023). Consistent with this, previous research has shown that parental growth mindset significantly influences and predicts the development of elementary school children's growth mindset (Chen and Liu, 2023; Lee et al., 2025). Thus, parents with a growth mindset regarding self-control are more likely to cultivate a similar mindset in their children. Importantly, this growth mindset in children can enhance their self-control abilities over time (Yuan et al., 2024). Given the proven positive impact of self-control on children's mental health (Kim et al., 2022; Moffitt et al., 2011), it is evident that parental growth mindset can indirectly benefit their children's mental health by promoting the development of self-control.

The study revealed that parenting self-efficacy and students' self-control play a chain mediating role between parental growth mindset and primary school students' mental health problems. This highlights the importance of parenting self-efficacy and students' self-control in the process of parental growth mindset influencing children's mental health problems. In line with previous studies, the family is the earliest and most significant socialization setting and has a significant impact on students' self-control (Cecil et al., 2012; Holmes et al., 2019; Richards et al., 2019). It is a key factor in this developmental process (Farley and Kim-Spoon, 2014; Kiss et al., 2014; Scully et al., 2020). This research further emphasizes the direct impact of parenting self-efficacy on students' self-control. In families with high levels of parenting self-efficacy, parents employ positive and constructive communication and conflict resolution methods. They help students to manage negative emotions and provide a supportive environment for practicing these behaviors, thereby promoting students' self-control (Glatz et al., 2024). When parents believe that children's self-control can be changed, they are more likely to be confident and adopt positive parenting practices (Muenks et al., 2015). Children learn self-control by observing and imitating their parents' behaviors (Guo, 2025). Thus, self-control is enhanced in children, which in turn improves their mental health.

In summary, this study confirms the significant influence of parental growth mindset on the mental health problems of primary school students and highlights the mediating roles of parenting self-efficacy and students' self-control. The chain-mediated mechanism reveals that parental growth mindset enhances parenting self-efficacy, which boosts students' self-control and ultimately promotes mental health. These findings offer a new insight into how families can impact the mental health problems of primary school students, providing a robust theoretical foundation for family-centered interventions. Because our parental measures center on self-control, effective interventions must target parental growth mindset about self-control, efficacy to cultivate it, and opportunities for children to practice it. Regarding the chain mediation path, although the indirect effect was small, such effects are clinically meaningful at the population level; when distributed across many families, even modest improvements can significantly reduce the public health burden of youth mental health problems. We interpret the chain mediation as a multi-step blueprint: targeting early links (growth mindset, parenting efficacy) can yield downstream returns in self-control and mental health, even if each individual path appears modest.

However, the current investigation has limitations that highlight potential areas for future research. One major limitation is the reliance on self-reported measures of parental growth mindset, which may introduce self-report bias and misalignment between parents' actual beliefs, parenting behaviors, and their reported mindsets (Li et al., 2023). A multi-informant approach that incorporates child reporting of parental mindsets alongside parent reports may provide a more accurate assessment of parents' mindsets. Specifically, child reports, compared to parent reports, may better reflect how children perceive their parents to behave in daily life (Haimovitz and Dweck, 2016; Schiffrin et al., 2019). Future research would benefit from adopting such a multi-informant approach to enhance the validity of the assessment by capturing the children's perspective, which is directly linked to their developmental experiences. Second, a significant limitation concerns the representativeness of the sample. Participants were recruited via convenience sampling from only two public schools in urban Beijing, which substantially limited the external validity of the study. Findings may not generalize to rural areas, private schools, or other regions in China with different socioeconomic and educational contexts, which could potentially limit their applicability to disadvantaged populations. Future research should employ stratified random sampling across multiple areas (urban, suburban, and rural) and school types (public, private) to enhance generalizability and test the boundary conditions of our model. Finally, an essential limitation of the design is that mental health problems were assessed only at T3, which precludes controlling for the baseline of mental health problems. While the temporal design (with predictors and mediators measured prior to the outcome) supports the hypothesized directional pathways, future research should include mental health assessments at T1 and T2 to strengthen causal inferences.

## 6 Conclusion

This longitudinal study examined the influence of parental growth mindset on the mental health problems of Chinese primary school students over time, as well as the potential mechanisms involved. The results demonstrated that parental growth mindset significantly negatively predicted students' mental health problems, with parenting self-efficacy and students' self-control serving as key chain mediators. These findings have important theoretical implications for educational and family-related research and provide practical guidance for creating a positive family environment to improve the mental health of primary school students.

## Data Availability

The raw data supporting the conclusions of this article will be made available by the authors, without undue reservation.
